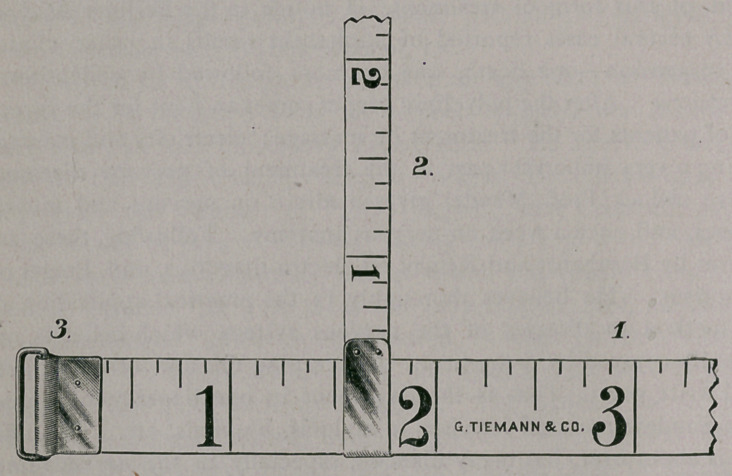# An Improved Tape Measure

**Published:** 1890-04

**Authors:** William C. Krauss

**Affiliations:** Niagara University, Buffalo, N. Y.; 176 Franklin Street


### New Instruments.

### AN IMPROVED TAPE MEASURE.

By WILLIAM C. KRAUSS, M. D., Niagara University, Buffalo, N. Y.

AMONG neurologists, perhaps, no symptom is more important and significant than muscular atrophy or wasting. The key-note of diseases affecting the anterior columns of gray matter of the cord, and of peripheral nerves, it is often improperly noted and erroneously measured.

The custom of comparing the two sides by sight or by touch, in many cases permits of errors which may be of serious moment in reaching a prognosis or diagnosis. The employment of the ordinary tape measure, a step approaching accuracy, also permits of discrepancies. The practice of taking measurements at the lower, middle, and upper third of the extremities may be sufficiently exact for one measurement on one side, but when double measurements are necessary for comparison, or when successive measurements are required, this mode is also inadequate, inasmuch as it is very improbable that the tape will be applied at exactly the same place as before.

To correct this difficulty, Messrs. Geo. Tiemann & Co., of New York, have made for me a tape measure which permits of the greatest accuracy possible, and the absolute exclusion of guess-work in using it.

It is particularly adapted to the measurement of the extremities, and consists of a tape (1) thirty-six inches long and one-half inch wide. The English scale is graduated on one side and the metric scale on the other. The head is supplied with a swivel (3), through which passes the free end of the tape, permitting of uniform tension, greater accuracy in reading, and of its being held with one hand.

The second tape (2) is eighteen inches long and one-quarter inch wide, and is provided with a sliding head through which the first tape passes. This tape is, therefore, at right-angles to, and movable upon, the first tape. It is also graduated after the English and metric scales. The object of this tape is to ascertain at what distance from a certain fixed bony point the first tape has been applied, so that on succeeding occasions the measurement may be taken at the same point. To illustrate: If the tape (1) be applied to the arm at a distance of five inches from the internal condyle of the humerus (reckoned by means of tape 2), it is obvious that on succeeding occasions, or in comparison of the two extremities, the tape (1) must be applied at exactly the same point, thus excluding all possible chance of error.

I believe this tape to possess certain points of value to neurologists, surgeons, and those intent upon accuracy and precision in their observations.

176 FRANKLIN STREET.

## Figures and Tables

**Figure f1:**